# Innovative gene engineering strategies to address tumor antigen escape in cell therapy

**DOI:** 10.1186/s12967-025-07259-8

**Published:** 2025-11-05

**Authors:** Yuning Chen, Siyue Niu, Yan-Ruide Li, Lili Yang

**Affiliations:** 1 Department of Microbiology, Immunology & Molecular Genetics, University of California Los Angeles, Los Angeles, CA 90095 USA; 2 Department of Bioengineering, University of California Los Angeles, Los Angeles, CA 90095 USA; 3 Molecular Biology Institute, University of California, Los Angeles, CA 90095 USA; 4 Eli and Edythe Broad Center of Regenerative Medicine and Stem Cell Research, University of California Los Angeles, Los Angeles, CA 90095 USA; 5 Jonsson Comprehensive Cancer Center, David Geffen School of Medicine, University of California Los Angeles, Los Angeles, CA 90095 USA; 6 Parker Institute for Cancer Immunotherapy, University of California Los Angeles, Los Angeles, CA 90095 USA; 7 Goodman-Luskin Microbiome Center, University of California Los Angeles, Los Angeles, CA 90095 USA

**Keywords:** Tumor antigen escape, Immunotherapies, CAR-based cell therapy, Unconventional t cell therapy; multi-specific cARs, Adoptive cell transfer

## Abstract

Tumor antigen escape limits the durability of antigen-specific immunotherapies, particularly chimeric antigen receptor (CAR)-based treatments. Malignant cells evade detection through six routes: antigen mutation or alternative splicing, impaired antigen processing, lineage switching, membrane redistribution, trogocytic epitope masking, and CAR-induced shielding during autologous manufacture. First noted in blood cancers, these tactics increasingly appear in solid tumors, where heterogeneity and immune suppression exacerbate escape. Emerging countermeasures broaden or restore antigen recognition: multi-specific modalities (dual/tandem CARs, bispecific engagers, adaptor CARs), logic-gated synNotch circuits, antigen-upregulating mRNA vaccines and epigenetic drugs, and non-conventional effectors such as invariant natural killer T (iNKT), gamma delta T (γδ T), and mucosal-associated invariant T (MAIT) cells. Collectively, these advances signal a shift toward adaptable, off-the-shelf, biomarker-guided platforms designed to keep pace with tumor evolution and achieve escape-resistant immunity.

## Background

Tumor antigen escape remains a significant barrier to the long-term success of antigen-specific immunotherapies, particularly chimeric antigen receptor (CAR)-based approaches. By altering, downregulating, or concealing target antigens, tumor cells can evade immune detection and resist therapeutic pressure, leading to disease relapse and treatment failure. Six major mechanisms underlie this phenomenon: genetic mutations or alternative splicing of antigen genes, deficits in antigen processing, lineage plasticity, antigen redistribution, epitope masking via trogocytosis, and contamination-related masking during autologous CAR-engineered T (CAR-T) cell manufacturing. These escape routes are not only prevalent across hematologic malignancies but are also increasingly recognized in solid tumors, where heterogeneity and immunosuppressive microenvironments further complicate effective targeting. Given this complexity, next-generation therapeutic strategies are being actively developed to counteract tumor antigen escape. These include multi-targeting CAR constructs (e.g., dual and tandem CARs), Bi-specific T cell engagers (BiTEs), adaptor molecule platforms, and synthetic circuit engineering like synthetic Notch (synNotch). Simultaneously, efforts to enhance antigen visibility—via mRNA vaccines or epigenetic modulators—and the introduction of unconventional immune effectors such as invariant natural killer T (iNKT), gamma delta T (γδ T), and mucosal-associated invariant T (MAIT) cells, offer additional layers of resilience against tumor evasion. This review explores the molecular underpinnings of antigen escape and highlights the most promising therapeutic advances designed to overcome it. Rather than cataloging approaches, we present a comparative, decision-oriented framework that links each escape mechanism to ranked engineering levers prioritized by near-term translational plausibility. We distill these comparisons into each section and conclude with a clinical translation roadmap, providing practical guidance for trial design, manufacturing, and patient selection.

## Biology of tumor antigen escape

**Fig. 1:**
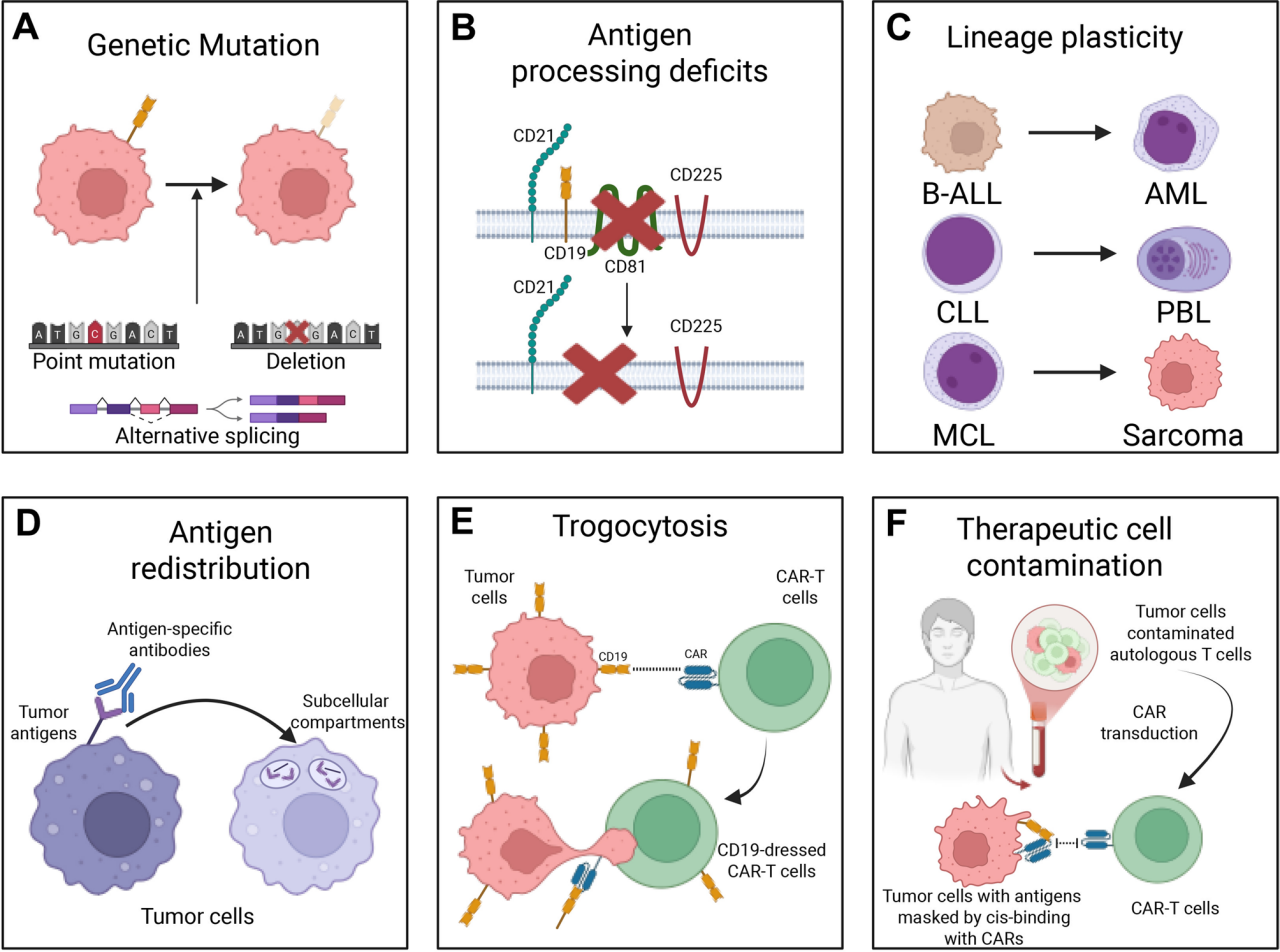
Mechanisms of tumor antigen escape from CAR-based immunotherapies. Tumor cells evade CAR-engineered immune cells through six key mechanisms. A. Genetic mutation: point mutations, deletions, or alternative splicing of antigen genes (e.g., CD19) reduce or eliminate surface expression, impairing CAR recognition. B. Antigen processing deficits: loss of chaperones like CD81 or regulators like NUDT21 disrupts proper antigen maturation and membrane localization. C. Lineage plasticity: tumor cells shift lineage identity (e.g., B-ALL to AML), downregulating target antigens and escaping detection. D. Antigen redistribution: antigens internalize into subcellular compartments upon binding, reducing surface availability for CAR engagement. E. Trogocytosis: antigens are transferred from tumor to CAR-T cells, depleting tumor antigen density and triggering CAR-T fratricide. F. Therapeutic cell contamination: tumor cells transduced during CAR-T manufacturing can mask antigens via cis-binding, evading immune attack. These escape routes contribute to therapy resistance and highlight the need for improved CAR strategies

Tumor antigen escape is one of the main hurdles to overcome by CAR-engineered cell therapies to achieve better tumor killing efficacy. Specifically, tumor antigen escape can be understood as a mechanism where tumor cells evade immune system, or in this case, therapeutic cells by losing or significantly downregulating the expression of specific tumor antigens that the therapeutic cells would normally recognize. There are six strategies that tumor cells adopt to achieve tumor antigen escape, specifically including antigen gene mutations, deficits in antigen processing, lineage switching, antigen redistribution, epitope masking, and trogocytosis, ultimately leading to resistance against targeted therapies (Fig. 1A–F).

Genetic alterations such as point mutations, deletions, and alternative splicing of tumor antigen genes can disrupt immune recognition, enabling immune evasion. In B-cell acute lymphoblastic leukemia (B-ALL), CD19-targeted CAR-T cell therapy selects for tumor cells expressing CD19 splice variants lacking critical epitopes. Specifically, the Δexon-2 variant lacks the extracellular epitope of CD19, whereas the Δexon-5,6 variant eliminates the transmembrane domain, both significantly reduce the surface presentation of CD19 on tumor cells, hence rendering CAR-T therapy ineffective [1–3]. Beside B-ALL, tumor antigen escape via antigen mutation or alternative splicing also manifests in other hematologic malignancies. Specifically, a biallelic loss of the G-protein-coupled receptor class 5 member D (*GPRC5D*) gene, due to local chromosomal deletion, was identified in a patient with recurrent plasmacytoma post GPRC5D-targeted CAR-T therapy [4]. In T-cell acute lymphoblastic leukemia (T-ALL), frameshift or missense mutations in exons one to three of CD7 were associated with antigen-negative relapse following anti-CD7 CAR-T therapy [5]. In solid tumors, antigen mutations also contribute to therapeutic resistance. For instance, *HER2* exon 16 deletion correlates with trastuzumab resistance in breast cancer, while the V600E mutation in *BRAF* confers resistance to vemurafenib in melanoma [6, 7].

Other than the altered/missing expression of tumor antigens, deficits in antigen processing can result in antigen escape as well. When treating acute lymphoblastic leukemia with BiTE, one of the patients was reported loss of CD81, a chaperon protein that form a complex with CD19, CD21, and CD225 to govern the maturation and transport of CD19 [8, 9]. In addition to CD81, nudix hydrolase 21 encoded by *NUDT21*, a protein that regulates the polyadenosine tailing and stability of CD19 mRNA, was found in elevated expression level that resulted in CD19-negative relapses in B-ALL patients after treatment with anti-CD19 CAR-T cells or blinatumomab [9]. Emerging work suggests several ways to restore surface target display. For example, chemical chaperones, such as 4-phenylbutyrate and tauroursodeoxycholic acid, can stabilize folding and facilitate endoplasmic reticulum exit of misfolded client proteins, a generalizable approach when CD19 is retained in the endoplasmic reticulum due to folding or glycosylation defects [10–13]. Modulating the CD19–CD81 axis may help as well: experimental perturbations that restore CD81 function or enhance CD19–CD81 complex formation have improved CD19 surface expression in model systems [14]. For NUDT21-driven alternative polyadenylation of CD19 mRNA, genetic suppression of NUDT21 increases CD19 abundance via alternative polyadenylation control and re-sensitizes B-ALL/BLASTS to CD19 CAR-T or blinatumomab in preclinical models [15].

Lineage plasticity represents another escape mechanism, particularly in leukemia. A rare but documented phenomenon, lineage switching occurs when leukemic cells transition from a lymphoid to a myeloid phenotype in response to selective pressure. This transformation is largely facilitated by mixed-linkage leukemia (MLL) rearrangement [16]. For instance, in mixed-lineage leukemia (MLL) rearranged B-ALL, CAR19 therapy can induce a lineage switch to acute myeloid leukemia (AML), leading to loss of CD19 expression and therapy resistance [17, 18]. The MLL gene (*KMT2A*) undergoes translocations involving over 80 partner genes (e.g., *AFF1/AF4*, *MLLT3/AF9*, *MLLT1/ENL*, *MLLT10/AF10*, and *MLLT4/AF6*), encoding a histone methyltransferase that alters *HOX* gene expression, particularly *HOXA9*, which governs hematopoietic differentiation [19–22]. Consequently, loss of normal regulatory function facilitates lineage transformation. This phenomenon has been observed in various contexts, including B-ALL to AML, chronic lymphocytic leukemia (CLL) to plasmablastic lymphoma, mantle cell lymphoma (MCL) to sarcoma, and T-ALL to AML [23–25].

Mechanistically, *KMT2A* fusions juxtapose the KMT2A N-terminus to transcriptional elongation partners, which recruit the super-elongation complex and DOT1L to target loci [26, 27]. This drives H3K79 hypermethylation and sustained transcription of *HOXA*/*MEIS1* and other stemness programs, enforcing an early progenitor-like state and blocking normal B-cell differentiation [28, 29]. Menin and LEDGF/p75 act as critical chromatin tethers for *KMT2A* fusion complexes, making the menin-KMT2A interaction a key dependency [27, 30]. These epigenetic features collectively increase lineage plasticity, providing a mechanistic basis for the B-to-myeloid conversion observed after CD19-directed therapies in *KMT2A*-rearranged disease [31–33]. Clinically, this biology underlies the efficacy of menin inhibitors such as revumenib, now with supportive trial data and regulatory precedent, while DOT1L inhibition shows target engagement but modest single-agent activity, motivating combinations [34–36].

Rather than a complete loss of tumor antigen expression, antigen redistribution—where antigens relocate from the cell membrane to subcellular compartments—also contributes to immune evasion from therapeutic cells. For instance, in B-ALL cells co-cultured with CAR19, live microscopy reveals that CD19 clusters at the immune synapse, leading to its subsequent internalization [9]. Similarly, antibodies targeting HER2, CD20, FLT3, EGFR, CD10, CD22, and prostate-specific membrane antigen (PSMA) have been shown to induce internalization of their respective antigens upon binding. This process undermines the efficacy of antibody-based treatments such as BiTEs or antibody-dependent cellular cytotoxicity (ADCC), which depend on the sustained surface presentation of tumor antigens to engage immune cells like CAR-T cells and natural killer (NK) cells [9, 37].

Another mechanism of antigen escape is trogocytosis, a process in which immune cells physically interact with tumor cells, forming an immunological synapse that causes the bidirectional transfer of membrane components (tumor antigens specifically) from tumor cells onto therapeutic cells. This process has two coupled consequences: it depletes antigen density on tumor cells, selecting for antigen-low variants that are harder to recognize, and decorates therapeutic cells with the same antigen, so they become inadvertent targets of one another, leading to fratricide [38, 39]. In addition, persistent low-level CAR engagement by the acquired antigen can sustain tonic signaling and accelerate exhaustion, further blunting efficacy [38, 39]. Specific instances of trogocytosis include the transfer of tumor antigens such as CD19, mesothelin, BCMA, and NKG2D ligands onto CAR-T or CAR-engineered NK (CAR-NK) cells. Additionally, immune checkpoint molecules such as HLA-G can be transferred from tumor cells to therapeutic cells, further contributing to immune evasion [40].

A rare yet clinically significant mode of tumor antigen escape arises from the inadvertent contamination of therapeutic cells with tumor cells during the manufacturing process of autologous CAR-T therapies. A resent study reported a case in which a patient with B-ALL relapsed due to the presence of CAR-transduced B cell leukemia cells [41]. These malignant cells were unintentionally transduced with the CAR during T cell production, leading to *cis*-binding of the CAR construct to the CD19 epitope on their surface. This interaction effectively masked CD19 from immune surveillance, rendering the tumor cells resistant to CAR-T cell therapy. While such occurrences are rare, they underscore the necessity of developing off-the-shelf CAR-based therapies, such as CAR-NK and CAR-engineered iNKT (CAR-iNKT) cells, where initial cells are sourced from healthy donors, to mitigate the risk of tumor cell contamination and enhance the safety of cell-based immunotherapies [41–43].

## Strategies addressing tumor antigen escape

### Diversification of tumor antigen-targeting receptors

#### Dual/multiple CARs

**Fig. 2:**
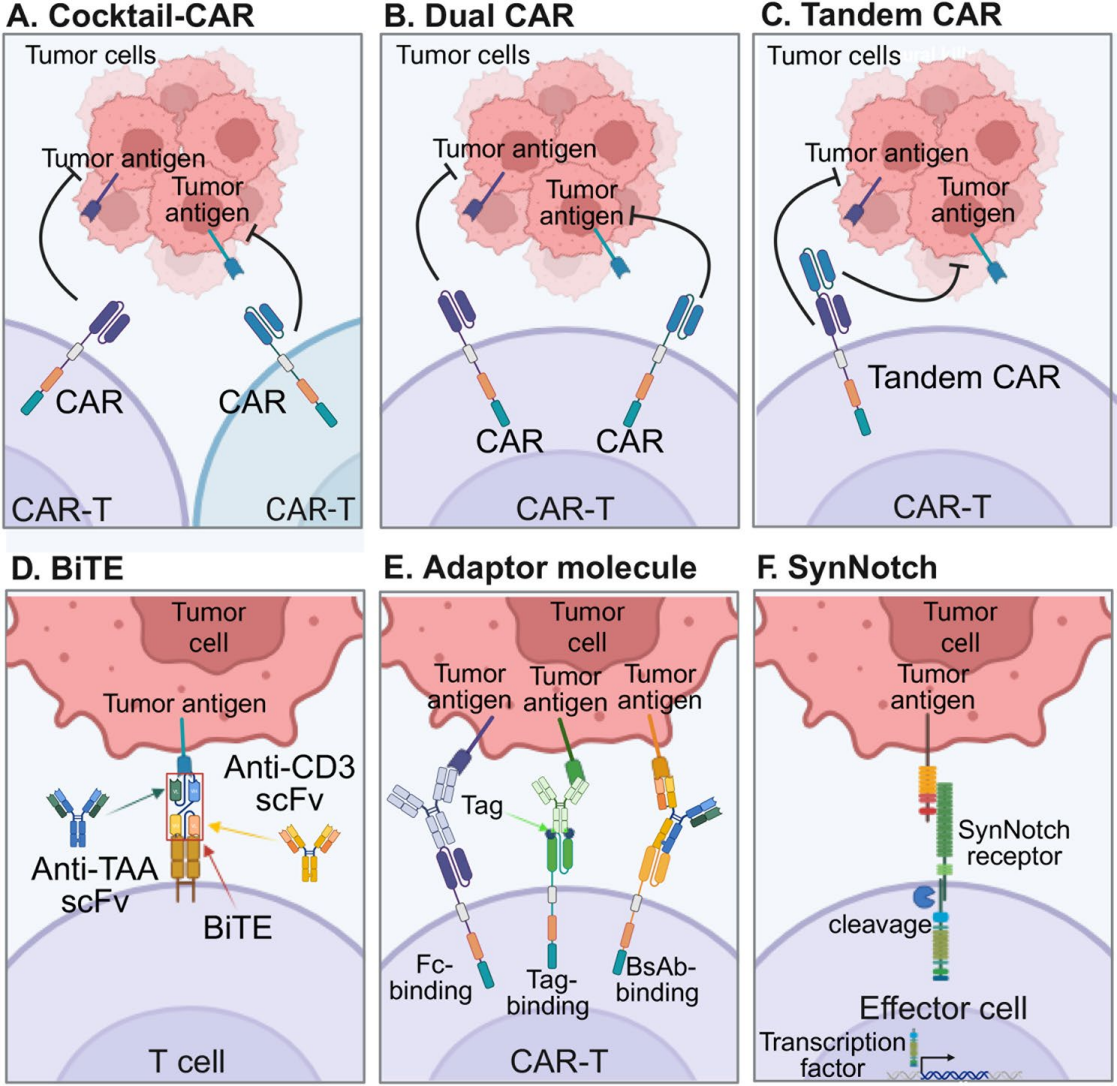
Strategies for broadening antigen recognition and overcoming heterogeneity/escape in adoptive cell therapy. A. Cocktail-CAR. Two (or more) monospecific CAR-T cell products, each directed to a different TAA, are infused together so that either CAR-T population can engage its corresponding antigen on heterogeneous tumor cells. B. Dual CAR. A single T cell is engineered to co-express two independent CAR constructs; ligation of either CAR with its respective TAA is sufficient to trigger effector activation. C. Tandem (bispecific) CAR. A single CAR molecule incorporates two linked scFvs, enabling simultaneous or independent binding to two TAAs through one receptor. D. BiTE. Soluble BiTE proteins combine an anti-CD3 scFv with an anti-TAA scFv, physically bridging conventional T cells to the tumor cell and initiating cytotoxicity without genetic modification. E. Universal CAR-T cells recognize an inert epitope (e.g., fc region, chemical tag, or small peptide) displayed on externally administered adaptor molecules—such as conventional antibodies, tagged antibodies, or bispecific antibodies, thereby allowing dose-tunable, on/off redirection toward multiple TAAs. F. SynNotch. Synthetic notch receptors sense a primary antigen, and then ligand-induced intramembrane cleavage releases a transcription factor that drives expression of user-defined genes, creating programmable logic gates that require sequential or combinatorial antigen inputs to activate effector functions

To address tumor antigen escape, combinatorial CAR-T cell strategies such as CARpool or cocktail-CAR and dual CAR approaches have been developed (Fig. 2A, B). In the CARpool model, two distinct single-input CAR-T cell products are manufactured separately and infused simultaneously or sequentially at different ratios [44, 45]. While clinical data show promise, with CAR19/22 T-cell cocktails achieving a 72.2% overall response rate and 50.0% complete response rate in refractory/relapsed B-cell non-Hodgkin lymphoma, parallel manufacturing of separate CAR T-cell populations remains logistically challenging [46]. Dual CAR designs overcome this limitation by engineering a single T cell population expressing two CARs through either co-transduction, bicistronic vectors, or tandem CAR constructs [46]. Mechanistically, dual CARs enhance efficacy through increased signal strength and improved T-cell/target cell interactions, resulting in synergistic rather than merely additive effects [46]. From a translational standpoint, dual targeting outperforms pooled single-target products in preventing antigen-loss relapse. For example, CD19/CD123 dual CARs better reduced CD19-negative escape compared to CAR “cocktails” in B-ALL models [47]. Clinically, there are high complete response rates for CD19/20 or CD19/22 dual-target approaches across B-cell malignancies, emphasizing the strategy’s advantage over single-antigen constructs [48]. These multi-targeting strategies not only expand the therapeutic arsenal but also provide more durable responses by addressing the heterogeneity of antigen expression and preventing escape-driven relapses [49].

Despite the higher tumor-killing efficacy, limitations of CAR cocktail and dual-targeting CARs remain. Parallel manufacturing of multiple single-input products increases cost, staffing, and turnaround time, and introduces coordination risk across apheresis, release testing, cryochain, and infusion scheduling. Multi-site programs report that co-administration and co-transduction approaches are more labor-intensive than building a single multi-target product [50, 51]. Moreover, co-transduction and bicistronic designs add genetic cargo, which can lower vector titer, reduce transduction efficiency, and create expression imbalance between the two receptors; this imbalance is linked to tonic signaling and impaired fitness, demanding careful spacer/linker and promoter tuning [52–54]. These constraints motivate process innovations such as point-of-care manufacturing to keep timelines clinically relevant [55].

#### Tandem CARs

Tandem CARs (TanCARs) represent an innovative strategy in CAR-T cell therapy, enabling dual antigen targeting within a single receptor construct (Fig. 2C). Like dual CAR-T and CARpool approaches, TanCARs follow an “OR” logic, allowing T cells to recognize and eliminate tumor cells expressing either of two target antigens. However, unlike these strategies, which require separate CAR constructs, TanCARs incorporate two single-chain variable fragments (scFvs) within a single CAR, facilitating simultaneous docking to two tumor-associated antigens (TAAs). This design enhances tumor recognition while maintaining the structural simplicity of a single transgene, reducing the risks associated with multiple gene insertions. A proof-of-concept study demonstrated the efficacy of TanCAR-T cells targeting HER2 and IL-13 Rα2 in glioblastoma, an aggressive and heterogeneous brain tumor. TanCAR-T cells exhibited superior anti-tumor responses compared to single-targeted CARs, effectively counteracting antigen escape. Notably, TanCARs outperformed dual CAR and CARpool approaches, potentially due to their ability to achieve superactivation when engaging both HER2 and IL-13 Rα2 simultaneously. Mechanistically, this heightened activation correlated with increased cytokine production, microtubule-organizing center (MTOC) polarization, and sustained cytotoxicity. Importantly, TanCAR-T cells did not exhibit elevated exhaustion markers such as TIM-3, LAG-3, or PD-1 following tumor engagement, suggesting preserved T cell function [56].

Beyond glioblastoma, TanCAR strategies have been explored in other malignancies. EGFRvIII/IL-13 Rα2 TanCARs have been tested in glioblastoma, while ErbB2/MUC1 TanCARs have been investigated for breast cancer. In hematologic malignancies, CD19/CD22 TanCARs have demonstrated preclinical efficacy against B-ALL, and a clinical study in 2020 evaluating CD19/CD20 TanCARs in refractory or relapsed B-cell lymphoma reported a 79% overall response rate, 71% complete response rate, and 64% progression-free survival rate [57–60]. In 2024, another phase I/II clinical trial reported that BCMA/CD19 tanCAR-T resulted in an overall response rate of 92% in patients with relapsed/refractory multiple myeloma [61].

While tanCAR-T cells have shown promises in overcoming tumor antigen escape, there are still challenges that remain to further extend the application of tanCAR-T. Specifically, engineering an optimal TanCAR construct requires careful consideration of spacer length, linker sequences, and scFv orientation, making its design significantly more complex than single-target CARs. Beyond scFvs, nanobody-based CARs offer improved receptor stability, lower immunogenicity, and strong antigen recognition: Cheng et al. developed CD70-specific nanobody-based CAR-T cells for AML that showed potent anti-leukemic activity and suggested combinatorial epigenetic upregulation of CD70 to enhance efficacy [62]. TanCAR constructs also increase chemistry, manufacturing, and controls (CMC) demands. For example, larger vector size can lower titer and transduction efficiency [63, 64]. Release testing must demonstrate potency aligned to mechanism: for dual-antigen CARs, activity against each target must be shown with lot-to-lot comparability according to FDA guidance. Additionally, the enhanced activation of TanCAR-T cells, while beneficial for cytotoxicity, may increase the risk of cytokine release syndrome (CRS) and neurotoxicity, particularly in solid tumors where antigen expression is not strictly tumor-specific. Targeting multiple antigens also raises concerns about on-target, off-tumor toxicity, potentially damaging healthy tissues that express low levels of the target antigens. Furthermore, while early preclinical and clinical data are promising, large-scale clinical trials are still needed to fully assess the long-term safety and efficacy of TanCAR therapy.

By overcoming these limitations through improved CAR design and safety mechanisms, TanCAR-T cells could offer a more effective and durable immunotherapy strategy, particularly for heterogeneous and antigen-escaping malignancies.

#### BiTEs

BiTEs are recombinant proteins engineered to concurrently activate cytotoxic T cells via the CD3 complex and direct them toward tumor cells (Fig. 2D). These molecules are comprised of two scFvs, one targeting the CD3 receptor on T cells, and the other targeting a TAA [65]. This dual specificity enables BiTEs to physically bridge T cells and tumor cells, triggering T cell activation and subsequent perforin/granzyme-mediated tumor cell apoptosis [66, 67]. Since BiTEs function independently of TCR specificity and MHC restriction, they can enhance T cell-mediated tumor killing by overcoming a key immune evasion strategy—MHC downregulation [66]. Furthermore, the incorporation of co-stimulatory molecules like CD28 and CD2 optimizes BiTE-mediated antitumor efficacy [68, 69].

A clinically successful example of BiTEs is blinatumomab, the first FDA-approved BiTE for treating ALL. It functions by linking binding domains for the B cell-specific antigen CD19 and the invariant CD3ε subunit of the TCR expressed on all T cells [66, 70]. The feasibility of blinatumomab in the clinical setting can be attributed to several characteristics. First, confocal microscopy studies have confirmed that blinatumomab can induce the formation of structurally normal immune synapses, which are essential for effective cytotoxic T lymphocyte (CTL) activity [71]. Directing T cells to recognize CD19 improves the proliferation of T cells and triggers the secretion of proinflammatory cytokines, including IL-2, IFN-γ, TNF-α, IL-4, IL-6, and IL-10 [72]. Notably, blinatumomab demonstrates exceptionally potent T-cell-mediated cytotoxicity, with half-maximal activity observed at concentrations as low as 10–100 pg/ml [73]. Furthermore, due to its single-chain structure, blinatumomab can be efficiently produced in large quantities using a robust purification strategy, resulting in a stable monomeric formulation suitable for clinical application [73].

Given the advantages of BiTEs, they also possess limitations that require further research. One major challenge is tumor antigen escape, where tumor cells downregulate or lose expression of the target antigen, leading to impaired tumor cell recognition. For example, in a clinical trial of blinatumomab, two out of four relapses were associated with the emergence of CD19^-^ tumor cells [74, 75]. The frequency of regulatory T cells (Tregs) can diminish the efficacy of BiTEs as well. Tregs suppress the immune system by expressing negative regulatory cell surface receptors and inhibitory soluble mediators, thereby reducing BiTE-induced immune response [76, 77]. Duell et al. demonstrated that ALL patients who responded to blinatumomab had lower Treg levels compared to non-responders, suggesting that Treg depletion may enhance the therapeutic efficacy of blinatumomab [78, 79]. Since BiTEs activate cytotoxic T cells to eliminate tumor cells, the upregulation of immunosuppressive markers such as PD-L1 on tumor cells can inhibit T cell activation and reduce therapeutic efficacy. In a clinical case, an increase in PD-L1-expressing cells was suspected to contribute to blinatumomab resistance in ALL, highlighting the role of adaptive immune resistance mechanisms in limiting BiTE effectiveness [80]. In addition, extramedullary relapse presents a challenge, as BiTEs like blinatumomab have limited ability to penetrate sites such as the central nervous system (CNS).

Overall, BiTEs represent an important advancement in cancer immunotherapy. Continued research in combination therapies, antigen selection, immune modulation, and delivery optimization will be pivotal in unlocking the full potential of BiTEs for a broader range of hematologic and solid tumors. By addressing these limitations, BiTE-based therapies may achieve greater durability and efficacy, ultimately improving outcomes for patients with resistant or relapsed malignancies.

#### Adaptor molecule

Adaptor molecule-based CAR-T cell therapies introduce an additional layer of modularity to conventional CAR designs by redirecting T cells not directly to tumor antigens, but to intermediary adaptor molecules that bridge tumor cells and CARs. Three major classes of adaptor systems have emerged (Fig. 2E). Fc-binding adaptor CARs leverage antibodies targeting tumor antigens, using CAR constructs engineered with Fc-binding extracellular domains, such as CD16, to recognize the Fc region of therapeutic antibodies [81]. This strategy benefits from the broad availability of clinically approved antibodies; however, variability in antibody glycosylation may affect CAR engagement, necessitating glyco-engineering [82]. Moreover, non-specific binding to circulating IgG poses a risk of off-tumor toxicity, although excessive IgG may paradoxically enhance tumor targeting under certain conditions [81, 83, 84]. Tag-binding adaptor CARs employ engineered extracellular domains that recognize chemically or genetically appended tags on tumor-targeting molecules. Platforms include biotin-binding domains (e.g., streptavidin), fluorescein-tagged antibodies, yeast or human-derived peptides, leucine zipper-based zipCAR systems, and SpyCatcher CARs that form covalent bonds with SpyTag-modified adaptors [85]. Key barriers include the potential immunogenicity of non-human tag-binder pairs, the challenge of maintaining switchability without sacrificing potency as affinity are tuned, and ensuring sufficient adaptor delivery and residence time in tumors [85–88]. Finally, bispecific antibody (bsAb)-binding CARs use extracellular domains (e.g., FRα, EGFRvIII, Cripto-1) that interact with bispecific adaptors bridging CAR-T cells and tumor cells [89]. The adaptor approach offers critical advantages, notably a molecular safety switch enabling dynamic modulation of CAR activity by titrating adaptor availability and the potential to develop universal CARs independent of tumor antigen heterogeneity. Nevertheless, challenges including immunogenicity, adaptor binding affinity optimization, and maintaining switchability without compromising potency must be addressed to fully realize the therapeutic potential of adaptor CAR systems.

#### SynNotch receptor & synthetic circuit

One of the principal challenges facing engineered cell therapies is the dynamic and heterogeneous nature of tumor antigen expression, which often leads to antigen escape and therapeutic failure. Unlike conventional CARs or TCRs that primarily regulate input recognition, synNotch receptors offer modular control over both antigen sensing and downstream gene expression. This dual-level control enables programmable, context-specific cellular behaviors that are finely tuned to environmental cues.

The synNotch system builds upon the natural Notch signaling mechanism, wherein ligand engagement induces intramembrane proteolysis and release of the Notch intracellular domain, which then translocates to the nucleus to modulate gene expression (Fig. 2F). Synthetic versions repurpose this architecture by engineering the extracellular domain to recognize a desired antigen, typically using scFvs [90–92] and replacing the intracellular domain with synthetic transcriptional regulators capable of driving expression of user-defined genes [93, 94]. Notably, synNotch circuits are functionally orthogonal to endogenous signaling pathways and to each other, allowing multiple independent synNotch modules to operate within a single cell. This modularity facilitates complex computations, including Boolean logic gating, spatial patterning, and multicellular signaling cascades [95].

This versatility has been exploited in several preclinical cancer models. For instance, Choe et al. designed synNotch receptors targeting the glioblastoma-specific neoantigen EGFRvIII or the CNS-restricted antigen MOG, which triggered expression of CARs directed against additional tumor-associated antigens. In intracerebral patient-derived xenograft models exhibiting heterogeneous EGFRvIII expression, synNotch-regulated CAR-T cells outperformed conventional CAR-T cells by selectively targeting more homogeneous, though less tumor-specific, antigens. This architecture mitigated tonic CAR signaling, preserved a naïve or stem cell–like memory phenotype, and prevented T cell exhaustion [96]. Similarly, synNotch circuits developed by Witzen et al., in which ALPPL2-specific synNotch receptors induce CARs targeting mesothelin or HER2, demonstrated superior tumor control in xenograft models of solid cancers compared to constitutively expressed CARs [97].

Beyond direct tumor targeting, synNotch platforms have been engineered to endow therapeutic cells with the capacity to sense disease-associated biomarkers or small molecules and secrete therapeutic payloads in response, effectively functioning as smart delivery systems in vivo [98, 99]. Lupo et al. further extended the therapeutic reach of synNotch by modifying NK cells derived from iPSCs to recognize CD155—an immunosuppressive ligand expressed by glioblastoma cells. Upon engagement, the synNotch receptor suppressed CD73, an ectoenzyme critical for adenosine-mediated immune suppression, thereby enhancing NK cell cytotoxicity, increasing T cell infiltration, and reducing immunosuppressive macrophage populations within the tumor microenvironment (TME) [100].

Clinical translation of synNotch-based therapies is underway, with a Phase I trial (E-SYNC) currently evaluating autologous EGFRvIII-specific synNotch T cells that induce dual CAR expression against EphA2 and IL13Rα2 in patients with EGFRvIII^+^ glioblastoma. Despite its promise, synNotch also presents technical limitations. Ligand-independent activation (LIA) can arise from overexpression of synNotch receptors, resulting in off-target gene expression. To address this, enhanced synNotch (esNotch) variants incorporating hydrophobic intracellular linkers have been developed, reducing LIA by over 14-fold while preserving activation fidelity [92]. Additionally, synNotch receptors are inherently dependent on mechanical force generated by surface-bound ligands, rendering them ineffective for detecting soluble cues or mediating responses in environments characterized by transient ligand interactions, such as neuronal synapses [101, 102].

Practical constraints include payload size, timing dependencies of synNotch-driven CAR induction, and ligand-independent “leak”, which together argue for circuit-function release assays that capture licensing kinetics and basal activity, in line with FDA potency/comparability guidance [63, 92, 96, 103, 104]. Clinically, the key risks are mis-activation in antigen-low normal tissues (on-target, off-tumor) and insufficient activity in highly heterogeneous tumors. Mitigation includes AND-gated designs tuned to indication-specific co-expression maps, locoregional delivery where appropriate, and pre-defined retargeting triggers when on-treatment biomarkers reveal antigen drift. Contemporary reviews of logic-gated immunotherapies emphasize these themes and the need for harmonized analytics and multicenter validation to move beyond single-center proofs of concept [105–107]. In practice, AND-gate strategies used by synNotch-primed CARs are being rationalized with tumor co-expression maps and single-cell datasets to reduce off-tumor activation while preserving efficacy in heterogeneous lesions [106, 108].

### Alternative therapeutic cell population with multiple mechanisms of action

#### iNKT cells

NKT cells represent a unique subset of lymphocytes that bridge innate and adaptive immunity by co-expressing a semi-invariant TCR and NK cell markers such as NKp46 and CD161. Among them, iNKT cells are the dominant population, characterized by an invariant TCR α-chain (Vα14-Jα18 in mice, Vα24-Jα18 in humans) that recognizes glycolipid antigens presented by the non-polymorphic, MHC class I-like molecule CD1d [109, 110]. Unlike conventional T cells restricted by polymorphic MHC molecules, iNKT cells’ CD1d restriction grants them broad allogeneic potential and enables recognition of conserved glycolipid antigens across individuals [111, 112].

**Fig. 3:**
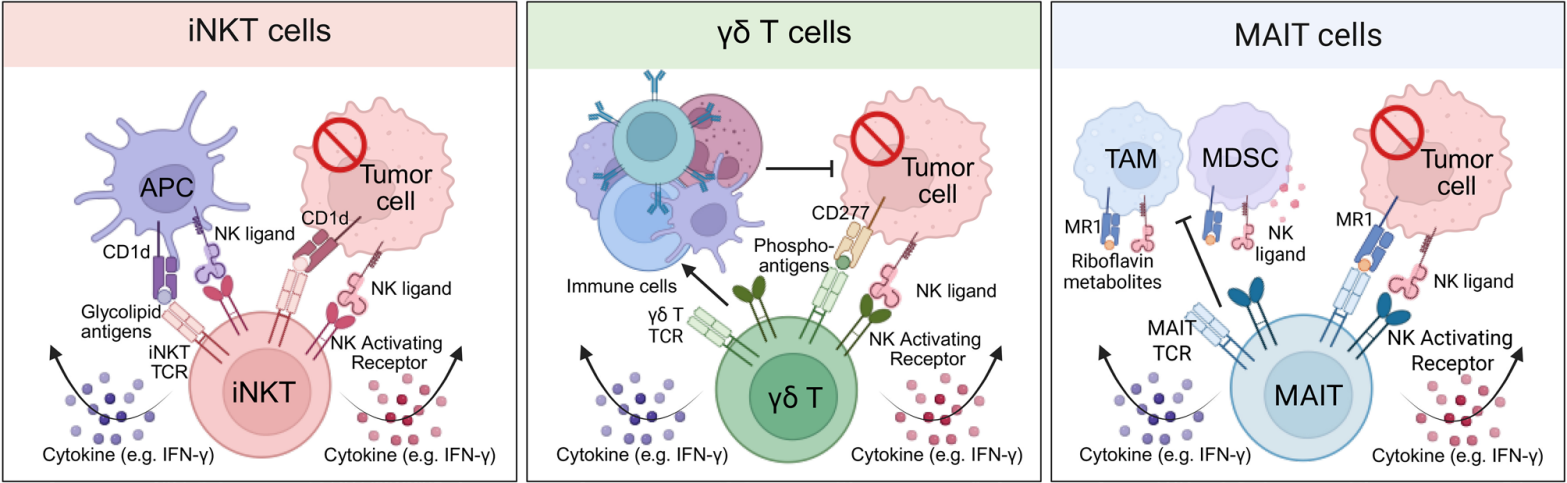
Unconventional T-cell subsets that deploy multiple recognition pathways to counter tumor-antigen escape. A. iNKT cells. iNKT cells recognize glycolipid antigens presented by the non-polymorphic CD1d molecule on APCs or tumor cells, and simultaneously engage NK-activating receptors. Dual activation drives perforin/granzyme-mediated cytolysis of CD1d^+^ tumor cells and TAMs while unleashing rapid Th1-type cytokine release (e.g., IFN-γ). These cytokines recruit and license NK cells, dendritic cells, and conventional T cells, enabling indirect killing of CD1d^−^ tumor variants. B. γδ T cells. γδ T cells detect non-peptidic phosphoantigens presented by CD277 family molecules through their γδ TCR, and co-activate via NK receptors that bind their ligands on tumor targets. This MHC-independent recognition triggers direct lysis of malignant cells and potent cytokine secretion. The released IFN-γ and TNF further activate other immune cells, providing indirect, cytokine-mediated antitumor effects with minimal risk of GvHD. C. MAIT cells. MAIT cells use a semi-invariant TCR to sense riboflavin-metabolite antigens presented by MR1 on tumor cells, TAMs, and MDSCs. In concert with NK-activating receptors, they execute cytotoxicity and secrete pro-inflammatory cytokines, reshaping the immunosuppressive tumor microenvironment

iNKT cell activation can occur via multiple mechanisms—either indirectly, through cytokines or CD1d^+^ antigen-presenting cells (APCs) such as dendritic cells (DCs), macrophages, or B cells, or directly, by recognition of tumor-associated glycolipid antigens presented by CD1d-expressing tumor cells [113–117] (Fig. 3A). This versatility is critical in tumor surveillance, especially in contexts where tumor cells downregulate MHC molecules or present stress-induced ligands. However, tumor cells can evade immunity by downregulating CD1d. To overcome this, administration of glycolipid agonists such as α-galactosylceramide (α-GalCer) has been shown to restore and boost iNKT cell-mediated antitumor activity by enhancing CD1d-mediated presentation and stimulating robust IFN-γ responses [118–121].

Once activated, iNKT cells deploy diverse mechanisms to exert antitumor effects: direct cytolytic killing via perforin/granzyme or through NK-like receptors (e.g., NKG2D), recruitment and activation of innate and adaptive effector cells, remodeling of the immunosuppressive TME, and the generation of long-term immune memory [122]. Notably, in tumors such as neuroblastoma, iNKT cells can kill CD1d^+^ tumor-associated macrophages (TAMs) and convert M2-like suppressive macrophages into pro-inflammatory M1-like macrophages, thereby mitigating TME-mediated immune suppression [114, 123–127]. Given their potent immunostimulatory and cytotoxic potential, iNKT cells are being harnessed in cancer immunotherapy. While early approaches using free α-GalCer had limited efficacy, attributed to the low basal frequency of iNKT cells in humans, subsequent strategies involving adoptive transfer of ex vivo expanded iNKT cells or α-GalCer-pulsed DCs have shown improved outcomes in clinical trials, including increased IFN-γ production and iNKT cell expansion [122, 128–130].

Among the most promising advances is the development of CAR-iNKT cells. Unlike conventional CAR-T cells, CAR-iNKT cells exhibit triple targeting through their CAR, invariant TCR, and NK-like receptors, offering broader and more flexible tumor recognition. Importantly, CAR-iNKT cells have demonstrated reduced risks of CRS and graft-versus-host disease (GvHD) [131, 132]. A phase I clinical trial evaluating anti-CD19 CAR-iNKT cells in relapsed/refractory B-cell malignancies has reported encouraging early results, including one complete response and one partial response—both without CRS or GvHD—underscoring the clinical potential of CAR-iNKT cells in overcoming tumor immune escape (NCT03774654). Additionally, CAR-iNKT cells have been shown to remodel the immunosuppressive TME by depleting CD1d^+^ TAMs and myeloid-derived suppressor cells (MDSCs). This dual activity enables them to target both tumor cells and the TME, resulting in superior tumor control compared with conventional CAR-T cells, particularly in solid tumors such as ovarian cancer, renal cell carcinoma, and glioblastoma [133–135].

By leveraging their unique recognition patterns, rapid cytokine production, and capacity to shape the immune microenvironment, iNKT cells—and especially CAR-modified variants—stand out as versatile agents capable of addressing key challenges in tumor immunoevasion, such as antigen loss and TME suppression.

#### γδ T cells

γδ T cells have gained increasing attention in cancer immunotherapy for their distinct antigen recognition capabilities and unconventional modes of action that set them apart from conventional αβ T cells. Structurally, γδ T cells express a T cell receptor composed of γ and δ chains, enabling them to recognize a broad array of antigens independently of MHC molecules [9, 136]. This MHC-unrestricted recognition equips γδ T cells with a unique advantage in targeting tumor cells that have undergone immune evasion via antigen loss or MHC downregulation, a common mechanism of resistance seen in solid tumors and hematologic malignancies. In contrast to αβ T cells, which typically require both antigen presentation and co-stimulatory signals for full activation, γδ T cells can mount a cytotoxic response with a single activating stimulus, underscoring their functional flexibility [137]. Some subsets, such as Vδ2 T cells, even act as professional antigen-presenting cells, facilitating broader immune responses and enhancing cross-talk with other immune compartments.

γδ T cells mediate antitumor immunity through several mechanisms: direct cytotoxicity via perforin and granzyme release, secretion of pro-inflammatory cytokines like IFN-γ and TNF, and engagement of NK-like receptors, including NKG2D, to recognize stress-induced ligands (Fig. 3B). Additionally, their MHC-independence drastically lowers the risk of GvHD, positioning them as an attractive allogeneic platform for adoptive immunotherapy. However, tumor-induced immunosuppression, coupled with the functional heterogeneity of γδ T cell subsets and their variable tissue tropism, poses barriers to their therapeutic consistency and persistence within the TME.

Building on their intrinsic antitumor properties, γδ T cells have been genetically engineered with CARs to enhance tumor specificity and cytotoxic potential. CAR-γδ T cells combine the innate-like surveillance and rapid effector function of γδ T cells with the precision targeting of CAR constructs. Preclinical models and early-phase clinical trials have demonstrated promise in treating B-cell lymphoma, glioblastoma, ovarian, and colorectal cancer [112, 138, 139]. Yet, several obstacles remain—most notably, the challenge of subset heterogeneity, limited in vivo persistence, and the need for efficient gene transfer techniques tailored to γδ T cells [140–142]. As researchers work to optimize expansion protocols and refine CAR design, the clinical utility of CAR γδ T cells will continue to evolve, offering a powerful new avenue for treating tumors that evade conventional T cell therapies.

#### MAIT cells

MAIT cells are an emerging force in cancer immunotherapy, offering a unique arsenal against tumor antigen escape. Structurally, MAIT cells express a semi-invariant TCR—composed of Vα7.2-Jα33 paired with a restricted β chain repertoire—that recognizes riboflavinderived microbial metabolites presented by the non-polymorphic MR1 molecule [143–146]. Their MR1-restricted antigen recognition is complemented by MR1-independent activation pathways involving cytokines such as IL12 and IL18, enabling a broad and flexible response [143, 147–149]. This antigen recognition independence from MHC not only allows MAIT cells to bypass tumor mechanisms that evade peptide presentation but also minimizes the risk of GvHD in allogeneic settings.

Upon activation—whether MR1dependent or cytokine-driven—MAIT cells rapidly proliferate and deploy multiple effector mechanisms: they release cytotoxic mediators such as perforin and granzyme B, secrete pro-inflammatory cytokines including IFN-γ and TNF, and engage NK-like receptors such as NKG2D to target stressed or transformed cells [150] (Fig. 3C). They also support immune coordination by enhancing dendritic cell and conventional T cell anti-tumor responses, and modulating tumor-infiltrating myeloid cells like TAMs and MDSCs [151]. These combined strategies render MAIT cells resilient to tumor antigen loss, enabling continued tumor surveillance even when conventional targets are absent.

To further harness this intrinsic versatility, novel strategies are emerging that genetically engineer MAIT cells as CAR-MAIT therapies. CAR-MAIT cells offer triple-layered targeting—via CAR, MR1-restricted TCR, and NK-like receptors—providing robust coverage against antigen-heterogeneous tumors. Early preclinical studies report efficient in vitro tumor cell killing, and MR1-restriction plus MHC-independence should favor off-the-shelf allogeneic use with reduced GvHD risk [143]. While optimization of CAR design, manufacturing protocols, and strategies to overcome exhaustion remain areas for refinement, the MR1-centric biology and innatelike cytotoxic breadth of MAIT cells position them as a compelling candidate to counter tumor antigen escape in nextgeneration immunotherapeutic platforms.

### Upregulate antigen expression/presentation on tumor cells

**Fig. 4:**
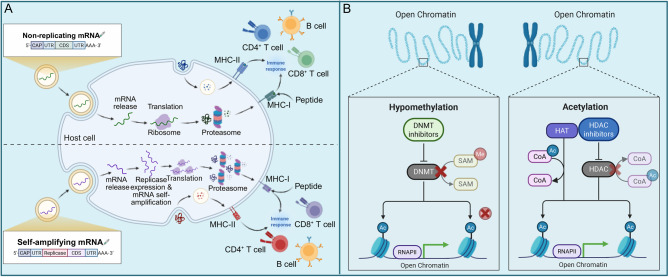
Strategies to upregulate antigen expression on tumor cells. A. Schematic representation of non-replicating and SAM mRNA vaccines in tumor antigen upregulation. Non-replicating mRNA vaccines deliver antigen-encoding transcripts directly for translation, while SAM vaccines enable prolonged antigen expression through self-replication. B. Epigenetic modulation of tumor antigen expression. DNMT inhibitors restore the expression of TAAs suppressor genes by reducing DNA methylation, whereas HDAC inhibitors promote gene transcription by inducing histone acetylation, leading to higher chromatin accessibility and anti-tumor gene expression

The efficacy of antigen-directed immunotherapies largely depends on the stable and sufficient expression of cancer testis antigens (CTAs) and TAAs on malignant cells. However, tumor heterogeneity and immune selection pressure often result in antigen downregulation or loss, leading to immune escape and therapeutic resistance. Strategies to upregulate antigen expression on tumor cells are therefore critical for optimizing tumor targeting and sustaining immune-mediated cytotoxicity (Fig. 4).

#### mRNA vaccine

mRNA vaccines have emerged as a promising platform for introducing tumor antigens into the body and activating specific anti-tumor immune responses [152]. Unlike conventional cancer vaccines relying on proteins or peptides, mRNA vaccines work by delivering the transcripts that encode one or more tumor-associated or tumor-specific antigens (TSAs) into host cells, primarily APCs, wherein the cytoplasm, these mRNA molecules are translated into antigens, which are subsequently presented on the surface of APCs via MHCs, thereby initiating and amplifying anti-tumor immune responses [153]. After administration, translated tumor antigens are degraded by endogenous pathways and presented to CD8^+^ cells by MHC-I molecules to trigger anti-tumor cytotoxicity [152, 154]. In contrast, a portion of the vaccine transfects nucleated cells, such as muscle cells and neutrophils, where intracellularly expressed antigens are subsequently released via exocytosis and taken up by APCs. These exogenously derived antigens are mainly presented by MHC-II molecules to CD4^+^ cells, which, in turn, enhance the activation of CD8^+^ T cells and B cells, thereby inducing both cellular and humoral immune responses against tumors [152, 155].

mRNA vaccines can be categorized into two major types: non-replicating and self-amplifying (SAM) vaccines (Fig. 4A) [156]. Non-replicating mRNA vaccines consist of full-length mRNAs encoding the targeted antigens, equipped with a 5’ cap structure and a 3’ poly(A) tail [157]. SAM vaccines are derived from positive-sense single-stranded RNA viruses, in which genes encoding the antigens of interest replace the original structural protein-encoding genes responsible for forming infectious viral particles with RNA replication machinery unchanged [153, 157]. Both types of vaccines have been tested in clinical trials. In a clinical study (NCT00831467) of the non-replicating prostate cancer vaccine CV9103, antigen-specific T cells were detected in approximately 80% of prostate carcinoma patients, regardless of their HLA background, with few adverse effects associated with the vaccine [158]. Similarly, a clinical trial investigating a SAM vaccine for the treatment of advanced or metastatic carcinoembryonic antigen (CEA)-expressing malignancies (NCT00529984) demonstrated the activation of antigen-specific effector T cells, highlighting the potential of SAM vaccines in eliciting robust immune responses against tumors [153, 159]. Although non-replicating mRNA vaccines have been more extensively studied in clinical trials for cancer treatment, the SAM platform offers a significant advantage over non-replicating mRNA vaccines by enabling prolonged and high-yield antigen production from a remarkably low-dose vaccination [153, 160–162]. This self-amplification mechanism enhances immunogenicity and may improve vaccine efficacy while reducing the required dosage.

mRNA vaccines represent a promising way to address tumor antigen escape. However, several challenges remain, including mitigating the effects of tumor heterogeneity, overcoming the immunosuppressive TME, optimizing administration routes, and identifying reliable biomarkers to predict treatment responses [163, 164]. Further research is necessary to address these limitations and enhance the efficacy of mRNA-based cancer immunotherapy [165, 166].

#### Chemical agent-induced antigen overexpression

Chemical agents can be employed to upregulate anti-tumor gene expression, thereby enhancing the efficacy of cell-based therapies. Epimutations, including hypermethylation and epigenetic silencing of tumor suppressor genes, contribute to the etiology of human cancers. Unlike DNA mutations, which are passively inherited through DNA replication, epimutations require active maintenance, as they are inherently reversible [167]. This dynamic nature provides an opportunity for therapeutic intervention using small-molecule inhibitors that target epigenetic modifications.

One strategy targeting epimutations is DNA methyltransferase (DNMT) inhibitors (Fig. 4B). By interacting and covalently binding to the catalytic site of DNMTs and disrupting their enzymatic activity, these nucleoside analogs lead to the degradation of DNMTs directly and reactivate silenced genes [168–170]. One type of DNMT inhibitor is 5-aza-2’-deoxycytidine, such as decitabine, which can be directly incorporated into DNA [171, 172]. As a unique cytosine analog, decitabine was initially recognized as a therapeutic agent for hematologic malignancies, including myelodysplastic syndromes (MDS) and CML, where it reactivates anti-tumor genes like p15^INK4b^, HIC1, p21^CIP1^, and p57^KIP2^ and upregulates CTAs like SPAN-Xb [173–176]. Although the hepatotoxicity associated with DNMT inhibitors has restricted their application in solid tumors, optimized dosing strategies have provided new opportunities for their potential therapeutic use [177, 178]. For example, decitabine-induced DNMT degradation has been discovered to restore the expression of tumor suppressor genes, enhance immune recognition, and increase the expression of TAAs associated with non-small cell lung cancer and neuroblastoma, including MAGE-A1, NY-ESO-1, and SSX, which are otherwise epigenetically silenced without decitabine [179–181]. Decitabine has been evaluated in clinical trials for both hematologic and solid malignancies, with its development reaching phase III trials for hematopoietic cancers and phase II trials for solid tumors. Decitabine has been evaluated in clinical trials for both hematologic and solid malignancies, with its development reaching phase III trials for hematopoietic cancers and phase II trials for solid tumors [172, 175, 178, 182–184].

Instead of the increase of DNA methylation, loss of lysine acetylation has been identified as the initial step in gene silencing [185]. Consequently, histone deacetylase (HDAC), which mediates the removal of acetyl groups from histones during this process, has emerged as a key therapeutic target [186, 187]. By maintaining a closed and compact chromatin structure, HDACs lead to the reduced gene expression [188, 189]. HDAC inhibitor prevents lysine deacetylation, thereby allowing histone acetyltransferases (HATs) to remain active, leading to sustained histone hyperacetylation and gene transcription (Fig. 4B). Several HDAC inhibitors have successfully passed clinical trials and received FDA approval, such as vorinostat and romidepsin [190]. For example, in cutaneous T-cell lymphoma, vorinostat upregulates the expression of IFN-γ, a cytokine that induces anti-tumor effect by upregulation of MHC molecules and activating CD8^+^ T cells [191–193]. However, HDAC inhibitors still present challenges due to their side effects, including nausea, vomiting, cardiac toxicity, and hematologic toxicity [194, 195]. Addressing these limitations through optimized dosing regimens, selective HDAC targeting, and improved drug formulations could enhance their clinical applicability.

In summary, chemical agents targeting epigenetic regulators offer a powerful approach to induce antigen overexpression in tumor cells, thereby enhancing the efficacy of cell-based cancer therapies. Besides DNMT and HDAC inhibitors, other epigenetic modulators, such as PMT inhibitors, are being actively investigated for their anti-tumor potential. By leveraging these epigenetic molecules, researchers are uncovering novel strategies to overcome tumor antigen escape and improve patient responses to immunotherapy.

#### Radiotherapy as an antigen-presentation enhancer

Radiotherapy can increase tumor visibility to cytotoxic lymphocytes by upregulating MHC class I and expanding the repertoire of peptides presented at the cell surface, a process described as immunogenic modulation and immunopeptidome broadening [196, 197]. Tailor et al. showed that irradiation induces differential regulation of antigen processing and presentation machinery, resulting in a global expansion of the immunopeptidome in tumor cells [198]. These antigen-presentation changes translate into better immune recognition and killing in vitro and in vivo, even where radiation alone is non-curative but primes tumors for immune attack [199]. Mechanistically, ionizing radiation induces DNA damage and cellular stress responses that elevate antigen-processing and presentation machinery and can increase presentation of tumor-specific peptides and neoantigens [198]. Radiation also releases tumor DNA that activates the cGAS-STING pathway within dendritic cells and tumor cells, driving type-I interferon programs that promote cross-presentation and prime tumor-specific CD8 T cell responses [200, 201].

Dose and fractionation influence these immune effects, with several studies indicating that hypofractionated and rationally fractionated regimens can enhance antigen presentation and systemic antitumor immunity compared with single large fractions, though the optimal schedule is context-dependent and shaped by stromal and immune composition [202–204]. For example, in relapsed large B-cell lymphoma, a short hypofractionated course such as 20–30 Gy in 5–10 fractions delivered to bulky sites in the week preceding anti-CD19 CAR-T has been associated with deeper metabolic responses at irradiated lesions and no excess CRS or immune effector cell-associated neurotoxicity syndrome (ICANS), consistent with a priming effect [205–208]. Clinical and translational studies support locoregional radiation as a priming strategy immediately before or during adoptive therapy, when MHC-I upregulation and peptide diversification are maximal [196, 209–211].

Careful field selection and dose constraints are important to amplify tumor-specific display while minimizing normal-tissue antigen upregulation that could increase on-target toxicity for antigen-directed agents [203]. Reviews and early clinical experiences combining radiation with CAR-T or other adoptive approaches emphasize the need for release and on-treatment biomarkers that confirm increased antigen presentation and for protocols that coordinate leukapheresis, manufacturing, and infusion with radiation timing [211, 212]. These practical considerations can convert radiation from a local cytotoxic tool into a system-level adjuvant that conditions tumors for more effective engagement by vaccines, engagers, and engineered lymphocytes [203, 213].

#### Innate agonists

Innate immune agonists provide another approach to enhance antigen expression and presentation on tumor cells. Agents targeting cytosolic DNA sensing and endosomal RNA receptors, most notably STING and TLR7/8 agonists, can reveal tumors that evade immune attack via poor antigen processing/presentation by inducing type-I interferon programs in dendritic cells, boosting antigen-processing machinery, MHC-I display, and cross-presentation to CD8 T cells [214, 215]. Mechanistically, cytosolic DNA generated by tumor stress activates cGAS-STING signaling in tumor cells and conventional type 1 dendritic cells (cDC1s), fueling IFN-stimulated gene programs and cross-priming of tumor-specific CD8^+^ T cells, and loss-of-function studies demonstrate that intact STING and cDC1 function are required for these antitumor effects [216].

Early-phase trials show that intratumoral STING agonists can be pharmacodynamically engaged in humans. Ulevostinag (MK-1454) given intratumorally alone or with pembrolizumab produced on-target biomarker induction including IFN-pathway genes with manageable safety and signs of antitumor activity in selected cohorts (NCT03010176 and NCT04220866) [217]. Phase I/Ib study of intratumoral E7766 demonstrated safety, pharmacodynamic activation, and preliminary efficacy with interferon-stimulated gene upregulation in the tumor microenvironment (NCT04144140) [218]. In parallel, recent translational studies with TLR7/8 agonists highlight DC activation, IFN-I-linked transcriptional responses, and enhanced cross-presentation capacity, supporting their use as priming agents in combination regimens [219, 220].

Multiple approaches have been employed for drug delivery. Since first-generation cyclic dinucleotides are transient with a limited membrane permeability, later delivery strategies concentrate STING signaling at the tumor/APC interface, including sustained intratumoral depots/hydrogels for prolonged local exposure, antibody-drug conjugates that ferry STING agonists to tumor or myeloid targets, and endoplasmic-reticulum-targeting constructs that amplify cross-presentation machinery [221–225]. Collectively, these approaches position innate agonists as practical priming tools that condition tumors for more effective engagement by vaccines, engagers, and engineered lymphocytes.

## Conclusions

Despite the transformative potential of antigen-targeted immunotherapies, tumor antigen escape remains a formidable obstacle that undermines treatment durability and contributes to relapse, particularly in both hematologic and solid tumors. Tumor cells can subvert immune recognition through diverse mechanisms—ranging from antigen mutation and alternative splicing to lineage switching, antigen redistribution, trogocytosis, and even manufacturing-related masking in autologous therapies. While a range of next-generation strategies have been developed—including multi-specific CAR constructs, bispecific engagers, synNotch circuits, antigen-upregulating agents, and alternative immune effectors such as iNKT, γδ T, and MAIT cells—challenges persist in translating these approaches into consistent, scalable, and safe clinical outcomes.

Recent readouts and reviews indicate steady progress: dual/logic-informed CAR designs and half-life–extended engagers are refining durability in selected hematologic settings, while early solid-tumor efforts show disease-stabilizing activity in tightly defined niches [226, 227]. For unconventional effectors (iNKT, γδ T, and MAIT cells), emerging trials underscore favorable trafficking/innate engagement with persistence optimization ongoing [112, 228]. Collectively, these trends support mechanism-guided modality selection and on-treatment antigen tracking to anticipate retargeting.

Key hurdles include the complexity of engineering and manufacturing multifunctional or modular CAR platforms, the risk of on-target, off-tumor toxicity from multi-antigen targeting, and the need to maintain T cell fitness and persistence in the immunosuppressive TME. Multi-specific CARs such as dual and tandem designs increase CMC complexity, requiring spacer and linker optimization to avoid tonic signaling and to preserve vector titer and expression balance across binders [229, 230]. Logic-gated circuits such as synNotch and engineered “AND” gates add genetic cargo and timing dependencies that necessitate circuit-function assays capturing licensing kinetics and leak before product release [231, 232]. For non-conventional immune cells like iNKT, γδ T, and MAIT cells, issues such as limited persistence, subset heterogeneity, and the lack of standardized expansion protocols require further optimization. Scalability will improve with banked donors, harmonized expansion protocols, and allo-evasion edits such as *TRAC*, *B2M*, or *CIITA* disruption to mitigate rejection and GvHD [233–235]. In addition, strategies such as mRNA vaccines or epigenetic modulators face challenges in targeted delivery, variable patient responses, and immune evasion from the tumor milieu.

Regulators emphasize demonstrable product consistency, clinically relevant potency, analytical comparability, and robust long-term risk management for cell and gene therapies [236]. Safety oversight continues to evolve, with standardized grading and management frameworks for CRS and ICANS adopted across trials and clinical practice [237]. Persistence monitoring and late-risk mitigation should follow FDA long-term follow-up guidance for gene therapy products, with predefined stop or de-escalation controls such as product withdrawal incorporated into study plans [236, 238].

Future directions will benefit from integrating synthetic biology with precision immunotherapy, using logic-gated circuits, feedback-controlled cytokine release, and antigen-sensing systems to fine-tune therapeutic responses. In the near term, programs should standardize biomarker stratification such as antigen density and embed longitudinal antigen monitoring and potency assays into early-phase trials. Then, leak-resistant synNotch, AND-gate designs and switchable/adaptor systems should enter multicenter studies, while off-the-shelf allogeneic platforms mature with persistence controls and harmonized CMC. Eventually, adaptive multi-antigen control, such as modular TanCAR/dual designs, logic circuits, or adaptor CARs, can be selected by patient-specific biomarker profiles with pre-planned retargeting at the first sign of escape. Continued development of off-the-shelf, allogeneic platforms and improvements in gene editing and cell expansion techniques will be crucial for broadening access and minimizing manufacturing-related risks. Lastly, combining these strategies with robust biomarker-driven patient stratification and real-time monitoring of antigen expression dynamics may unlock the full potential of personalized, escape-resistant immunotherapy. Overcoming antigen escape is not just a matter of targeting better—it requires reengineering the entire therapeutic paradigm to adapt, respond, and stay one step ahead of tumor evolution.

## Data Availability

No datasets were generated or analyzed during the current study.

## Abbreviations

CAR: Chimeric antigen receptor
BiTE: Bi-specific T cell engager
synNotch: Synthetic Notch
iNKT: Invariant natural killer T cell
MAIT: Mucosal-associated invariant T cell
B-ALL: B-cell acute lymphoblastic leukemia
GPRC5D: G-protein-coupled receptor class 5 member D
T-ALL: T-cell acute lymphoblastic leukemia
MLL: Mixed-linkage leukemia
AML: Acute myeloid leukemia
CLL: Chronic lymphocytic leukemia
MCL: Mantle cell lymphoma
PSMA: Prostate-specific membrane antigen
ATCC: Antibody-dependent cellular cytotoxicity
NK: Natural killer cell
CARB: CAR-positive leukemic B cell
PBL: Plasmablastic lymphoma
TanCAR: Tandem CAR
scFv: Single-chain variable fragment
TAA: Tumor-associated antigens
MTOC: Microtubule-organizing center
CRS: Cytokine release syndrome
TCR: T cell receptor
ALL: Acute lymphoblastic leukemia
CTL: Cytotoxic T lymphocyte
Treg: Regulatory T cell
CNS: Central nervous system
bsAb: Bispecific antibody
LIA: Ligand-independent activation
esNotch: Enhanced synNotch
APC: Antigen-presenting cells
DC: Dendritic cell
α-GalCer: α-galactosylceramide
TME: Tumor microenvironment
TAM: Tumor-associated macrophages
GvHD: Graft-versus-host disease
MHC: Major histocompatibility complex
MDSC: Myeloid-derived suppressor cell
CTA: Cancer testis antigens
TSA: Tumor-specific antigens
SAM: Self-amplifying
CEA: Carcinoembryonic antigen
DNMT: DNA methyltransferase
MDS: Myelodysplastic syndromes
CML: Chronic myeloid leukemia
HDAC: Histone deacetylase
HAT: Histone acetyltransferases
ICANS: Immune effector cell-associated neurotoxicity syndrome
cDC1: Conventional type 1 dendritic cells
CMC: Chemistry, manufacturing, and controls
